# Reliability and Validity of the Ethiopian Version of the Hospital Anxiety and Depression Scale (HADS) in HIV Infected Patients

**DOI:** 10.1371/journal.pone.0016049

**Published:** 2011-01-25

**Authors:** Ayalu Aklilu Reda

**Affiliations:** Department of Public Health, College of Health Sciences, Haramaya University, Harar, Ethiopia; Leicester University, United Kingdom

## Abstract

**Background:**

The hospital anxiety and depression scale (HADS) is a widely used instrument for evaluating psychological distress from anxiety and depression. HADS has not yet been validated in Ethiopia. The aim of this study was to evaluate the reliability and validity of the Amharic (Ethiopian language) version of HADs among HIV infected patients.

**Methods:**

The translated scale was administered to 302 HIV/AIDS patients on follow up for and taking anti-retroviral treatment. Consistency assessment was conducted using Cronbach's alpha, test-retest reliability using intra-class correlation coefficients (ICC). Construct validity was examined using principal components analysis (PCA). Parallel analysis, Kaiser's criterion and the scree test were used for factor extraction.

**Results:**

The internal consistency was 0.78 for the anxiety, 0.76 for depression subscales and 0.87 for the full scale of HADS. The intra-class correlation coefficient (ICC) was 80%, 86%, and 84% for the anxiety and depression subscales, and total score respectively. PCA revealed a one dimensional scale.

**Conclusion:**

This preliminary validation study of the Ethiopian version of the HADs indicates that it has promising acceptability, reliability and validity. The adopted scale has a single underlying dimension as indicated by Razavi's model. The HADS can be used to examine psychological distress in HIV infected patients. Findings are discussed and recommendations made.

## Introduction

The Hospital Anxiety and Depression Scale (HADS) is a widely used health related quality of life (HRQoL) instrument for measuring psychological distress. It was developed in 1983 by Zigmond and Snaith [1], [2] to screen and evaluate the presence and progression of clinically significant depression and anxiety in patients presenting at the general medical clinic. This brief scale has 14 items with half devoted to anxiety and half to depression. The anxiety has questions such as ‘I still enjoy the things I used to enjoy’ (item 2) for examining depression and; ‘I get a sort of frightened feeling as if something awful is about to happen’ (item 3) for examining anxiety. HADS aims to assess only the non-somatic aspects of psychological distress and as a result it does not have items that tap somatic symptoms of psychological distress.

Since its publication, the HADS has been translated into most of the European and some Asian languages, but very few published studies of adoption into the African languages exist [3]. Three reviews [4]–[6] and hundreds of primary studies have been conducted to investigate its psychometric properties. In many of the studies HADS has shown good acceptability, reliability and validity as indicated by good response rates (≥90%), high consistencies (Cronbach's alpha) ranging from 0.76 to 0.93 for the anxiety and 0.72 to 0.90 for the depression subscales, and good diagnostic/discrimination abilities [4], [5]. Factor analyses of the HADS commonly indicate the two dimensions suggested by the original authors and the three dimensional model of Watson et al [4], [6], [7]; followed by the four dimensional model of Anderson *et al* [8], [9] and Razavi's one factor model in few reports [10]–[12].

While it is known that psychological distresses are a recognized problem among HIV/AIDS patients and screening for them are important clinical goals [13], there are only very few studies that adopted the HADS in this patient group [14], [15]. The HADS has not been adopted into the Ethiopian languages. The aim of this study was to adopt the HADS into Amharic (the language of Ethiopia) and test its acceptability, reliability and validity among HIV/AIDS patients.

## Methods

### The questionnaire

The HADS is a questionnaire intended for the diagnosis and evaluation of anxiety and depression in non-psychiatric patients[1], [16], [17]. Anxiety and depression subscales are each represented by seven items. The items are rated on a four point Likert scale ranging from 0 to 3 giving maximum and minimum scores of 0 and 21 respectively for each subscale. Sub-scores on the anxiety or depression subscales ranging from 0 to 7 are considered normal; while 8 to 10 and 11 to 21 are considered ‘cause for concern’ and ‘probable cases of anxiety or depression’ respectively.

### Translation

The questionnaire was translated from English to Amharic by the author. The translated and the English versions of the HADS were then presented to health professionals working in the study area. The reviewers consisted of a panel of two experienced GPs, an internist, two nurses, a clinical psychologist, two psychiatric nurses, and a psychiatrist working at a teaching hospital. In addition, the scale was pretested on fifteen HIV infected patients and five non-patients where they were encouraged to comment on the acceptability and clarity of the items and the scale as a whole. The input of the patient and non-patient groups was also presented for the panel. The final translated items used for data collection were generated through consensus on the wording, clarity and cultural equivalence of items (refer to supporting file, File S1, for the translated scale).

### Data collection

Two nurses administered the HADs through face-to-face interviews after they completed practical training on the procedures of data collection and standardization of interviews. The scale was administered to a convenience sample of 302 HIV/AIDS consecutive patients taking antiretroviral treatment (ART) from April to May 2010 (the sample to item ratio is 22∶1 and is considered more than adequate [18]). In addition to items of the HADS, socio-demographic and clinical variables such as age, sex, CD4 count, WHO clinical stages (an indicator of severity of AIDS ranging from I, less severe; to IV, very severe AIDS stage) were incorporated. Recent results of the last two variables were abstracted from patient cards.

### Data analysis

Descriptive statistics and ceiling and floor effects were analyzed. Floor effects indicate ‘worst health scores,’ 21 for both dimensions of HADS and 42 for the total score; while ceiling effects indicate ‘best health scores,’ with a score of zero for both the subscale and total score.

Reliability was assessed using consistency and test-retest reliability analysis. Cronbach's alpha was used to indicate consistency reliability, where an alpha of 0.7 to 0.9 was considered good [19]. Test-retest reliability was examined by re-administering HADs after two weeks on the initially interviewed half of the sample. Test-retest reliability was assessed using intra-class correlation coefficients (ICC), where an ICC of more than 0.6 was considered satisfactory [19].

Correlation was examined using Pearson's correlation coefficients for ratio scale variables and Kendal's tau for WHO clinical stages (ordinal measure). Discriminant validity was examined by comparing the scores of participants in different categories of HIV/AIDS disease severity based on the WHO clinical stage and CD4 count quartiles using the Kruskal Wallis test.

Construct validity was analysed using exploratory factor analysis (EFA) using principal components analysis (PCA). To find the best fit to the data, orthogonal and non-orthogonal, as well as the non-rotated factor analysis were conducted. Factor loadings of more than 0.40 were considered satisfactory [18]. Findings of the PCA were further validated by split-half reliability analysis in which the findings of the factor structure on the whole data were repeated on random halves of the sample. In line with recommendations [20], [21], factors generated by the PCA were extracted as valid if at least two of the following criteria were met: (1) eigenvalues were more than the randomly generated factors from Horn's parallel analysis; (2) pass scree (Cattel's) test and; (3) eigenvalues of equal or more than unity [22], [23]. SPSS 15.0 was used for reliability and validity analysis, while Monte-Carlo PA software was employed for parallel analysis [24]. An alpha of less than or equal to 0.05 was considered significant.

### Ethical clearance

The author declares that no competing interests exist. The Research Ethics Committee of Haramaya University reviewed and provided ethical approval for the study. Each participant gave written consent. Participants who came back after two weeks for a second interview were refunded 10 birr ($1.50, purchasing power adjusted) to cover transportation expenses.

## Results

A total of 302 patients participated in the study (100% response rate). The mean age of the patients was 33.8 years (SD±8.4). Baseline characteristics are shown in table 1. From the 176 participants that expressed consent to come back for a second interview after two weeks, 144 (81.8%) returned. It took about 5–10 minutes to administer the HADS.

**Table 1:** Background and clinical characteristics of HIV/AIDS on antiretroviral treatment (ART)^a^.

Characteristic	N^b^	Percent
Sex		
Male	104	34.4
Female	197	62.4
Age (Mean, sd)	33.8	±8.4
Marital status		
Married	129	42.7
Single	67	22.2
Divorced	61	20.2
Widowed	41	13.6
Disease stage		
WHO stage I	28	9.3
WHO stage II	32	10.6
WHO stage III	214	70.9
WHO stage IV	28	9.3
CD4 count (Mean, sd)		
1^st^ Quartile	377.4	180.7
2^nd^ Quartile	388.1	206.7
3^rd^ Quartile	424.0	340.2
4^th^ Quartile	498.4	212.7
Over all	394.1	208.2

a N (%) unless indicated otherwise;

b N(%) is based on number of complete responses.

### Score distributions

There were no ‘floor’ effects for both subscales and the total score. However, there was modest amount of ‘ceiling’ effect on both subscales and the full scale as shown in table 2.

**Table 2:** Scores of participants at baseline assessment‡.

HADS	N	Mean (SD)	Median (range)	‘Floor’ effects, % n (worst health score)	‘Ceiling’ effects, % n (best health score)
Anxiety	302	4.0 (3.56)	3.00 (20)	0.00 (0)	16.90 (51)
Depression	302	3.98 (4.09)	3.00 (20)	0.00 (0)	23.20 (70)
Total	302	7.98 (7.15)	6.00 (20)	0.00 (0)	8.60 (26)

‡ Anxiety and depression are rated from 0 to 21 indicating best to worst health scores respectively; the total score is rated from 0 to 42 indicating best to worst health scores respectively.

### Reliability

There was good consistency between items, Cronbach's alpha for anxiety and depression subscales and the full scale were 0.78, 0.76 and 0.87 respectively. The ICC was 77.9% (95% CI 69.3-84.0), 86.1% (95% CI 80.7-90.0), and 84.0% (95% CI 77.6-88.4) respectively for anxiety and depression subscales and the full scale. Furthermore, in the first and second tests the anxiety and depression subscales, and the total scale correlated significantly with their corresponding counterparts well. Statistically significant correlations of 0.64, 0.76, and 0.72 were detected (p<0.01) for the anxiety and depression subscales and the full scale respectively.

### Construct and discriminat validity

The correlation between anxiety and depression subscales was 0.75 (p<0.000), while between the subscales and full scale it was 0.92 (p<0.000) and 0.94 (p<0.000) in the respective order. All the items correlated with the domain specific and full scores significantly (p<0.000). However, items correlated more to the domain to which they belonged (p<0.000). The total score versus item correlations had values lying in between the domain specific correlations (p<0.000) (table 3). There was poor correlation between the subscale and full scale scores, and WHO clinical stage (r <0.062, p>0.05) and CD4 counts (r<0.07, p>0.05) (table 3).

**Table 3:** Correlation of items with the anxiety and depression subscales and overall score of the HADS.

(Item no.) Items, subscales and full scale	HADS-A¥	HADS - D§	HADS- Tφ
(1) I feel tense or ‘wound up’ (A)	0.73	0.54	0.67
(3) I get a sort of frightened feeling as if something awful is about to happen (A)	0.65	0.42	0.57
(5) Worrying thoughts go through my mind (A)	0.62	0.47	0.58
(7) I can sit at ease and feel relaxed	0.71	0.62	0.71
(9) I get a sort of frightened feeling like ‘butterflies’ in my stomach (A)	0.60	0.42	0.54
(11) I feel restless as if I have to be on the move (A)	0.67	0.50	0.62
(13) I get sudden feelings of panic (A)	0.64	0.49	0.60
(2) I still enjoy the things I used to enjoy (D)	0.50	0.67	0.63
(4) I can laugh and see the funny side of things (D)	0.50	0.66	0.63
(6) I feel cheerful (D)	0.63	0.73	0.73
(8) I feel as if I am slowed down (D)	0.49	0.64	0.61
(10) I have lost interest in my appearance (D)	0.55	0.67	0.66
(12) I look forward with enjoyment to things (D)	0.36	0.57	0.50
(14) I can enjoy a good book or radio or a TV programme (D)	0.39	0.62	0.55
HADS – A	1		
HADS – D	0.75	1	
HADS – T	0.92	0.94	1
CD4 count	0.07	0.02	0.05

All correlations are significant at the 0.01 level (2-tailed) except CD4 count (p>0.05).

§ HADS – D, HADS depression subscale;

¥ HADS – A, HADS anxiety subscale; HADS – T,

φ HADS total score; A, anxiety; D, depression.

Neither the subscales nor the total score discriminated well between quartiles of the CD4 count (p>0.05) or between WHO stages of disease (p>0.05). The different groups had an almost similar mean score (<4.7 for subscales, <9.1 for the total score) except that more severe stages had a slightly higher but statistically non-significant score.

The principal components factor analysis (PCA) revealed a one factor model explaining 38.4% of the variation with an eigenvalue of 5.38, where all items loaded markedly onto this factor (table 4). The second factor explained 7.3% of the data with an eigenvalue of 1.02. This was not extracted as it was not above the randomly generated criterion eigenvalue from parallel analysis and did not fulfill the scree test criterion (table 5). The non-rotated factor produced the most explanation of the data and excellent loadings. The split half analysis also validated the findings of the whole sample PCA.

**Table 4:** Shows factor extraction decision that takes into account different criteria (n = 302)¥.

Factors	Eigenvalue	Total variance accounted for	Extraction Criteria				Decision to extract
			PA Random eigenvalue (SD)		Kaiser	Scree test	
1	5.38	38.4%	1.35 (0.04)	Yes	Yes	Yes	Yes
2	1.02	7.3%	1.27 (0.03)	No	Yes	No	No
3	0.96	6.8%	1.21 (0.03)	No	No	No	No

¥ The non rotated factor analysis provided the best fit to the data; Kaiser-Meyer-Oklin (KMO) sampling adequacy equals 0.92; All anti-image matrices measures of sampling adequacy (MSA) were greater than 0.90; Bartlett's test of sphericity, p<0.001; Yes, indicates criteria is fulfilled, No indicates otherwise. Kaiser recommends extracting factors with eigenvalue of ≥1; Scree (Cattel's) test recommends extracting factors above the elbow of the scree plot; PA, parallel analysis.

**Table 5:** Factor loadings of HAD scale items after factor analysis without rotation (n = 302)¥.

HAD Scale item	Factor 1	Factor 2
Anxiety sub-scale		
(1) I feel tense or ‘wound up’	**0.70**	0.06
(3) I get a sort of frightened feeling as if something awful is about to happen	**0.61**	−0.26
(5) Worrying thoughts go through my mind	**0.56**	−0.11
(7) I can sit at ease and feel relaxed	**0.61**	−0.17
(9) I get a sort of frightened feeling like ‘butterflies’ in my stomach	**0.59**	−0.47
(11) I feel restless as if I have to be on the move	**0.76**	−0.27
(13) I get sudden feelings of panic	**0.72**	−0.21
Depression sub-scale		
(2) I still enjoy the things I used to enjoy	**0.63**	−0.04
(4) I can laugh and see the funny side of things	**0.55**	**0.44**
(6) I feel cheerful	**0.66**	0.16
(8) I feel as if I am slowed down	**0.62**	0.29
(10) I have lost interest in my appearance	**0.47**	0.39
(12) I look forward with enjoyment to things	**0.63**	0.32
(14) I can enjoy a good book or radio or a TV program	**0.50**	0.06

¥ Bold indicates an item loading of 0.40 or more.

## Discussion

The findings indicate that the Ethiopian version of the HADS is an easy to administer instrument for measuring emotional distress. It showed good consistency between the items and high test-retest reliability. The HADS has one underlying factor as indicated by Razavi *et al.* [10], [11] and Chaturvedi [12]. This factor explained close to 40% of the variation within the data.

Given the 100% response rate, HADS seems to be well acceptable by HIV infected patients. The re-administration had also good response rate of 82.0%. The fact that patients were willing to come back for a second interview could indicate the minimal burden, as indicated by the limited length (only 5–10 minutes), administration of the test puts on patients; and aspects such as good face validity of the Amharic version of HADS. This is similar to findings from other studies. The difference in acceptability findings of other studies with this one mainly arises from the fact that we used interview-based administration of HADS. HADS takes even less time to administer (2–6 minutes) when self-administration method is used [5], [17]. The observed convenience in this study makes HADS an easy to use and time saving tool that could be used by non-psychiatric nurses and physicians alike in line with the aim of the developers of the scale [5], [17].

In this study HADS had very good consistency and test-retest reliability which is similar to findings in HIV/AIDS [14] and other patient groups [4], [5]. Comparison using Bjelland *et al.* [4] and Hermann's [5] review indicates that the Cronbach's alpha in this study is more than or equal to the highest reports of previous studies. This indicates good consistency between the items of the translated instrument.

HADS scores were not different across groups of participants based on groups of patients with different WHO disease stages or quartiles of CD4 count. Furthermore, it did not correlate well with both disease stage and CD4 count in a similar manner to the finding by Burgess *et al.* [25] and Savard *et al.* [14]. This could be because disease classifications are commonly somatic based, while HADS measures non-somatic emotional distress. This is not surprising according to a review by Hermann [5], where severity of disease as measured by tumor size or metastases for cancer; medical prognosis; and proximity to death were not positively related to scores on the HADS. In fact there are even reports of very low scores in severely ill and near death patients [5]. However, studies report that HADS has good discriminant validity when applied on non-somatically distinct groups with psychological distress [4], [5].

An interesting finding of this study is the unifactorial model of the Ethiopian version of HADS, similar to the reports of Razavi [10], [11] and Chaturvedi [12] where the items loaded markedly on the first factor. However, this is dissimilar to the common findings of bifactorial and trifactorial models of HADS [3]–[6]. In the literature, there seems to be disagreement as to the correct model of HADS. While the developers of the scale report a two factor model, other researchers [4]–[6] seriously contest this and come up commonly with three factors [26]–[28], and even up to four [9] and five factors [29].

The most disparate reports in the construct validity of the HADS are concerning the number of underlying dimensions or factors it possesses. Three possibilities or a combination thereof could explain this: the first would be that the dimensions of the HADS are actually either two or more than two; the second would be that the dimensions could differ from one patient (or socio-cultural) group to another; and the third, probably of not least importance and of which further discussion is made below, are methodological such as the factor extraction criterion employed [30]. The latter one is important when it comes to reports of the validation of HADS. Parallel analysis is considered the most reliable criteria for factor retention and outperforms Kaiser's and Catell's criteria [20], [21], however, it is underutilized by authors that validated the HADS. Hence, the fact that authors commonly used either Kaiser's criteria and/or the scree (Catell's) test for factor retention is a strong source of difference in findings. For instance, Karimova and Martin [29] used Kaiser's [22] criteria of eigenvalues above 1 when they reported 4–5 factors among pregnant women. In this manner other authors have also reported a bi-factorial model. This may also happen because of their expectation of a two factor model or due to their criteria of extraction [14], [31]. Another important source of difference between studies is socio-cultural differences that exist between populations where conceptions of anxiety, depression and emotional expression may differ. Furthermore, while most studies of HADS used self administration, this study used interview based questionnaire administration which is recommended for illiterate participants, this approach could contribute to the observed differences between this study and others.

Anxiety and depression scores are correlated and commonly coexist [30], [32]. Due to this, researchers contend that HADS may be measuring emotional distress or psychological disturbance in general rather than separate entities of depression and anxiety [5], [10], [12], [30]. However, even when the two dimensions of anxiety and depression are generated theoretically, it is reported that a practical overlap between the two is to be expected [5]. The findings of this study imply that caregivers need to focus more on generalized psychological distress than the separate entities of anxiety and depression. For the sake of convenience, the cut off (≥13 and ≥19 on the total score indicating levels of distress) suggested by Razavi *et al.* [11] may be employed until further case finding studies reliably estimate better cut offs based on specificity and sensitivity analyses.

### Limitations

This study has limitations. The study did not employ backward-forward translation method. It would have been more productive had this method been used. Despite this limitation, pre-test was done and the reviewers debated and revised the translation and wording of the Amharic version of the HADS rigorously before reaching the final version. Concurrent (diagnostic) validity was not assessed using gold standard (criterion) interviews or parallel measurements of anxiety and depression as there are no validated instruments for measuring anxiety or depression in Ethiopia. Had such instruments been used, the discriminatory validity analyses would have also been more dependable as these measurements would provide known groups of participants with a specific emotional distress. Due to this, the findings of discriminatory analysis need to be interpreted with caution.

### Conclusions

This preliminary validation study of the Ethiopian version of the HADs shows that it has promising acceptability, reliability and validity but not yet conclusive. The adopted scale has a single underlying dimension as indicated by Razavi's model. The HADS can be used to examine psychological distress in HIV infected patients. Additional studies need to be conducted to further explore the validity, reliability and case finding ability of this brief and easy-to-use scale not only in HIV/AIDS but also in other patient groups.

## Supporting Information

File S1 The Amharic version of the HADS.
(PDF)
