# Supplementary material for: Reliability and Validity of the Ethiopian Version of the Hospital Anxiety and Depression Scale (HADS) in HIV Infected Patients
Source: PLoS One. 2011 Jan 25;6(1):e16049. doi: 10.1371/journal.pone.0016049 (PMC3026786; doi:10.1371/journal.pone.0016049)
Supplement: File S1 — The Amharic version of the HADS. (PDF) [file pone.0016049.s001.pdf]

የጭንቀት እና የቁዘማ (ሀዘን) መገምገሚያ መጠይቅ

ስነልቦና ብዙ በሽታዎች ላይ የራሱ የሆነ ሚና እንዳለው ይታወቃል እናም ሀኪምዎ የሚሰማዎትን ስሜት ካወቀ በደንብ ሊረዱዎት ይችላሉ።

ባለፈው ሣምንት የተሰማዎን ትክክለኛ ስሜት መሠረት በማድረግ የጤና ባለሙያዉ ቀጥሎ ለሚጠይቁዎት ጥያቄ ተገቢውን መልስ ይስጡ።

በተቻለ መጠን ሲመልሱ ብዙ ጊዜ አይዉሰዱ፣ ምናልባትም እንደተጠየቁ ወዲያውኑ የመጣልዎት መልስ ትክክለኛ ስሜትዎን ሊገልፅ ይችላል።

1 የመጨነቅ ወይም የመወጠር ስሜት ምን ያህል ይሰማዎታል?

በጣም ብዙ ጊዜ	3	
ብዙ ጊዜ	2	
አልፎ አልፎ	1	
ምንም አይሰማኝም	0	

2 ቀደም ሲል ያስደሰቱዎ የነበሩ ነገሮች አሁን ምን ያህል ያስደስቱዎታል?

አሁንም እንደድሮው ያስደስቱኛል		0
ከድሮው ትንሽ ቀንሷል		1
በጥቂቱ ያስደስቱኛል		2
ጭራሽ አያስደስቱኝም		3

3 አንድ መጥፎ ነገር ሊያጋጥምዎ የተቃረበ የሚመስል የፍርሀት ስሜት ይሰማዎተል?

እጅግ በጣም ይሠማኛል	3	
በጣም ይሠማኛል	2	
በጥቂቱ ይሠማኛል	1	
ምንም አይሠማኝም	0	

4 መሳቅና የነገሮችን አስቂኝ ጎን ማየት ይችላሉ?

አብዛኛውን ጊዜ እችላለሁ		0
እንደድሮው ባይሆንም እችላለሁ		1
በጥቂቱ እችላሁ		2
ምንም አልችልም		3

5 ጭንቀትን የሚያጭሩ ሀሳቦች በአእምሮዎ ምን ያህል ጊዜ ይመላለሳሉ?

በጣም ብዙ ጊዜ	3	
ብዙ ጊዜ	2	
አብዛኛውን ጊዜ ባይሆንም አልፎ አልፎ	1	
አንዳንዴ ብቻ	0	

6 ደስተኛ ነዎት?

ምንም ደስተኛ አይደለሁም		3
ብዙ ጊዜ ደስተኛ አይደለሁም		2
ብዙም ባይሆን ደስተኛ ነኝ		1
አብዛኛውን ጊዜ ደስተኛ ነኝ		0

7 ተረጋግተው መቀመጥ እና ዘና ማለት ይችላሉ?

ሁሌም እችላለሁ	0	
አብዛኛውን ጊዜ እችላለሁ	1	
ብዙውን ጊዜ አልችልም	2	
ምንም አልችልም	3	

8 ስራዎን ሲያከናውኑ ወዘተ ፍጥነትዎ ምን ያህል የቀነሰ ይመስልዎታል?

እጅግ በጣም ብዙ ጊዜ	3
በጣም ብዙ ጊዜ	2
አልፎ አልፎ	1
ምንም አልቀነሰም	0

9 ሆድ አካባቢ የሚሰማ የመደንገጥ ወይም የመሸበር ስሜት ይሰማዎታል?

ምንም አይሰማኝም	0
አልፎ አልፎ	1
ብዙ ጊዜ	2
በጣም ብዙ ጊዜ	3

10 ለአለባበስዎ ትኩረትን መስጠት አቁመዋል?

አዎን ምንም ትኩረት እየሠጠሁ አይደለም	3
የምፈልገውን ያህል ትኩረት እየሰጠሁ አይደለም	2
ድሮ ከምሰጠው ትኩረት በጥቂቱ ያነሰ ትኩረትን እሰጣለሁ	1
ሁሌም የምሰጠውን ትኩረት እሰጣለሁ	0

11 አንድ ቦታ መሄድ ያለብዎ ይመስል ተረጋግቶ መቀመጥ ይቸገርዎታል?

በጣም ብዙ ጊዜ ይቸግረኛል	3
ብዙ ጊዜ ይቸግረኛል	2
ብዙም አይቸግረኝም	1
ምንም አይቸግረኝም	0

12 መጪ ነገሮችን በደስታ ይጠብቃሉ?

አዎ ሁሌም በተለመደው ወይም በድሮው መጠን እጠብቃለሁ	0
ከድሮው ወይም ከተለመደው በጥቂቱ ባነሰ መጠን እጠብቃለሁ	1
ከድሮው ወይመ ከተለመደዉ ባነሰ መጠን እጠብቃለሁ	2
ምንም በደስታ አልጠብቅም	3

13 በድንገት የመደንገጥ ወይም የመሸበር ስሜት ይሰማዎታል?

በጣም ብዙ ጊዜ ይሰማኛል	3
ብዙ ጊዜ ይሰማኛል	2
አልፎ አልፎ ይሰማኛል	1
ምንም አይሰማኝም	0

14 በሬዲዮ ወይም የቴሌቪዥን ኘሮግራሞች ራስዎን ያስደስታሉ?

አዎን ብዙ ጊዜ	0
ብዙም ባይሆን አዎ	1
አልፎ አልፎ	2
በጣም አልፎ አልፎ	3
